# Remnant Cholesterol and Residual Risk of Atherosclerotic Cardiovascular Disease

**DOI:** 10.31083/RCM25985

**Published:** 2025-02-20

**Authors:** Xi Li, Zhi-Fan Li, Na-Qiong Wu

**Affiliations:** ^1^Cardiometabolic Center, FuWai Hospital, National Center for Cardiovascular Diseases, Chinese Academy of Medical Science, 100037 Beijing, China

**Keywords:** remnant cholesterol, atherosclerotic cardiovascular disease, lipid-lowering drugs, major adverse cardiovascular events, angiopoietin-like protein 3, apolipoprotein C-III, proprotein convertase subtilisin/kexin type 9

## Abstract

Remnant cholesterol (RC) is increasingly recognized as a key target in the treatment of atherosclerotic cardiovascular disease (ASCVD), addressing much of the residual risk that persists despite standard therapies. However, integrating RC into clinical practice remains challenging. Key issues, such as the development of accessible RC measurement methods, the identification of safe and effective medications, the determination of optimal target levels, and the creation of RC-based risk stratification strategies, require further investigation. This article explores the complex role of RC in ASCVD development, including its definition, metabolic pathways, and its association with both the overall risk and residual risk of ASCVD in primary and secondary prevention. It also examines the effect of current lipid-lowering therapies on RC levels and their influence on cardiovascular outcomes. Recent research has highlighted promising advancements in therapies aimed at lowering RC, which show potential for reducing major adverse cardiovascular events (MACEs). Inhibitors such as angiopoietin-like protein 3 (ANGPTL3), apolipoprotein C-III (apoCIII), and proprotein convertase subtilisin/kexin type 9 (PCSK9) have demonstrated their ability to modulate RC and reduce MACEs by targeting specific proteins involved in RC synthesis and metabolism. There is a pressing need for larger randomized controlled trials to clarify the role of RC in relevant patient populations. The development of targeted RC-lowering therapies holds the promise of significantly reducing the high rates of morbidity and mortality associated with ASCVD.

## 1. Introduction

Atherosclerotic cardiovascular disease (ASCVD) remains the leading cause of mortality worldwide. Traditional risk factors contributing to ASCVD include dyslipidemia, hypertension, diabetes mellitus, and unhealthy lifestyles [1]. Managing elevated low-density lipoprotein cholesterol (LDL-C) levels is a cornerstone of lipid-lowering therapy and is essential for both primary and secondary prevention of ASCVD [2, 3]. However, a residual risk of ASCVD persists, even when optimal LDL-C levels are achieved and conventional risk factors are well-controlled. Lipoproteins, such as triglyceride-rich lipoproteins (TRLs), which are not typically addressed in standard lipid-lowering therapies, may explain this residual risk [4]. Epidemiological and genetic studies have shown that it is the cholesterol content of TRLs, rather than triglycerides (TGs) themselves, that significantly contributes to the initiation and progression of ASCVD [5, 6, 7]. Consequently, remnant cholesterol (RC) has emerged as a promising therapeutic target in preventing ASCVD.

## 2. Definition and Metabolism of RC

RC refers to the cholesterol fraction of TRLs and has significant implications for managing ASCVD. TRLs include very-low-density lipoprotein (VLDL) and intermediate-density lipoprotein (IDL) during fasting, as well as chylomicron remnants (CM-R) in the postprandial state. These lipoproteins are closely linked to triglyceride levels.

TRLs are synthesized through two primary pathways: the endogenous and exogenous routes. In the endogenous pathway, apolipoprotein (apo) B100 is assembled within hepatocytes, with the help of microsomal triglyceride transfer protein (MTTP), to form VLDL [8]. Subsequently, apolipoproteins CI, CII, CIII, and E bind to VLDL particles. The triglycerides within the VLDL core are hydrolyzed by lipoprotein lipase (LpL) into free fatty acids (FFAs), which are then transformed into IDLs and smaller, dense VLDLs [9]. These particles may evolve into low-density lipoprotein (LDL) via further action of LpL and hepatic lipase or be cleared directly by hepatocytes through receptor-mediated and independent pathways [10]. The exogenous pathway involves the small intestinal epithelium, where FFAs and glycerol are incorporated into chylomicron particles containing apoB48, facilitated by MTTP. These particles enter the circulation via the lymphatic system [11]. In the bloodstream, the core triglycerides of chylomicrons are hydrolyzed by LpL, yielding FFAs and leading to the formation of a smaller, cholesterol-enriched chylomicron remnant [9]. These remnants are cleared by hepatocytes through LDL receptor, LDL receptor-related protein 1 (LRP), or heparan sulfate proteoglycan (HSPG) pathways [12]. When TRL is overproduced or LpL function is impaired, incompletely lipolysed TRL lingers in the bloodstream. After lipolysis by cholesteryl ester transfer protein (CETP), TRLs can be reshaped into smaller size and more cholesterol-rich TRLs remnants [13].

LpL is pivotal in the metabolism of RC, and genetic mutations within LpL, such as the loss-of-function* Asp9Asn* and *Asn291Ser *variants, can elevate RC levels. Additionally, several LpL regulators, including apoCⅢ, angiopoietin-like proteins 3, 4, and 8, act as inhibitors of LpL, while apo A-V, apo C-II, lipase maturation factor 1 (LMF1), and glycosylphosphatidylinositol-anchored high-density lipoprotein binding protein-1 (GPIHBP1) activate it [14, 15].

**Fig. 1.**
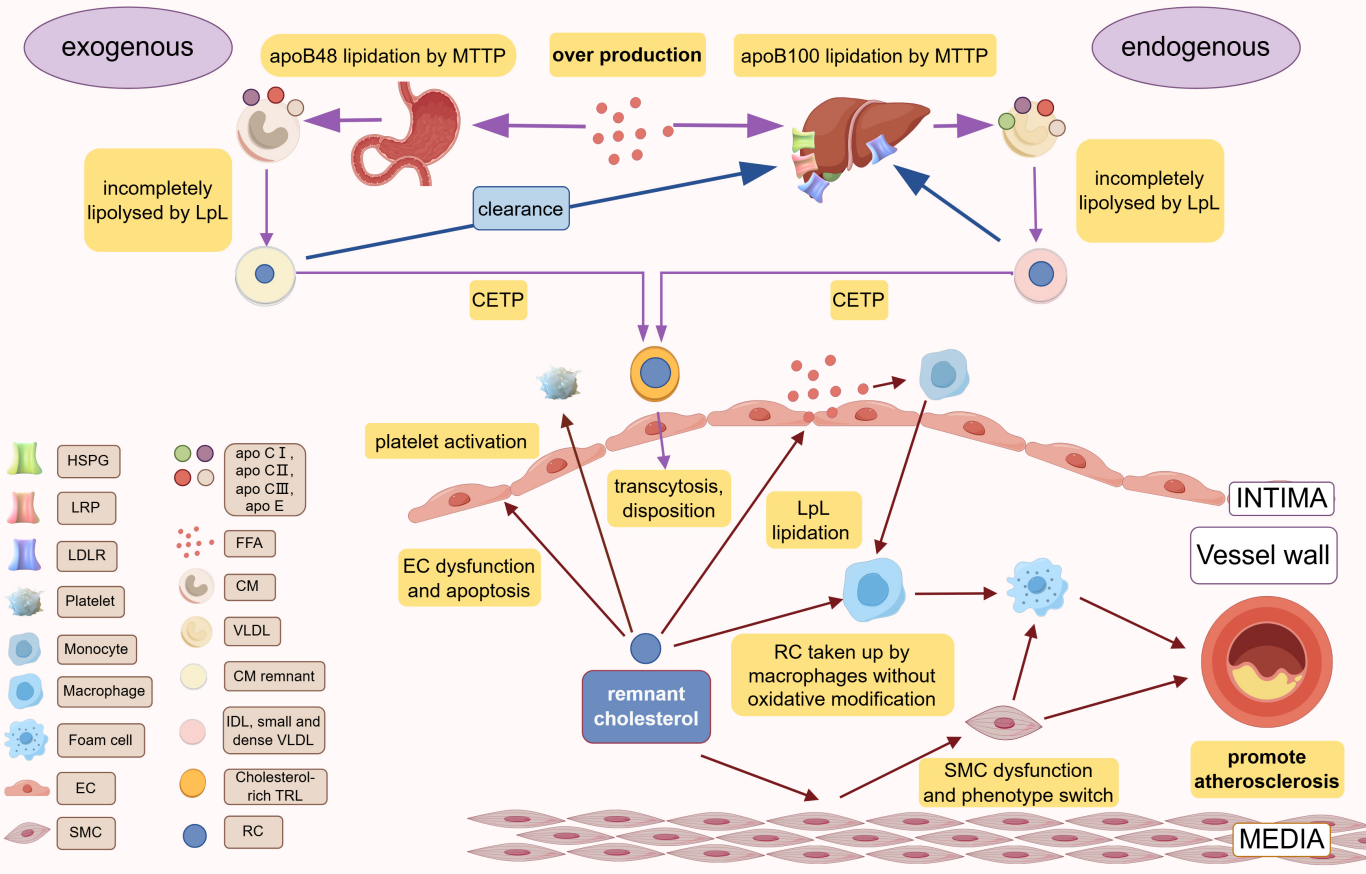
**Production, metabolism, and cardiovascular pathogenesis of RC**. Different species of apoB are lipidated by MTTP in the liver (apoB100) and small intestine (apoB48) to form VLDL and CM, respectively. In the blood circulation, incomplete lipolysis of LpL produces CM remnant and IDL, small and dense VLDL, which are exchanged their TG with cholesterol esters of TG through CETP to obtain the final products, cholesterol-rich TRLs. RC is the cholesterol component of cholesterol-rich TRLs. CM and IDL/small and dense VLDL are cleared by the liver via the various receptors (LDLR, LRP, HSPG). Over production of FFA or LpL dysfunction or abnormal hepatic receptor would cause the accumulation of RC. RC can lead to atherosclerosis via causing vascular endothelial cell dysfunction, causing chronic low-grade inflammation of the vessel wall via FFA released by LpL lipidation, promoting platelet activation. apo, apolipoprotein; IDL, intermediate-density lipoproteins; TRLs, triglyceride-rich lipoproteins; EC, endothelial cells; RC, remnant cholesterol; MTTP, microsomal triglyceride transfer protein; LDLR, low-density lipoprotein (LDL) receptor; LRP, LDL receptor–like protein; HSPG, heparan sulfate proteoglycans; FFA, free fatty acid; CM, chylomicron; VLDL, very low-density lipoprotein; SMC, smooth muscle cell; LpL, lipoprotein lipase; CETP, cholesteryl ester transfer protein; VLDL, very low-density lipoprotein. Created with Figdraw.com (https://www.figdraw.com/).

Due to their small size (<70 nm), remnant lipoproteins which contain RC readily penetrate arterial endothelial cells [14], potentially forming foam cells upon phagocytosis by arterial macrophages and myocytes. It can also induce endothelial dysfunction by releasing inflammatory cytokines such as tumor necrosis factor-alpha (TNF-α) and interleukin-1 beta (IL-1β), disrupting endothelium-dependent vasodilation, and fostering oxidative stress [7]. Furthermore, RC may contribute to chronic low-grade vascular inflammation and enhance thrombogenicity [13]. These mechanisms collectively contribute to the pathogenesis of atherosclerosis (Fig. 1).

Given the heterogeneity of RC, accurate measurement methods remain limited and RC levels are typically estimated either by calculation or direct measurement. The calculation method subtracts the sum of LDL-C and high-density lipoprotein cholesterol (HDL-C) from total cholesterol to estimate RC levels. Direct measurement techniques, such as ultracentrifugation, immunoseparation, and automated assays, provide more detailed insights.

Ultracentrifugation, or vertical automated profiling, separates lipoprotein classes and subclasses by concentration gradients, aggregating cholesterol from IDL and VLDL3 to determine RC levels [16]. Immunoaffinity techniques using mixtures of anti-apo B-100 and anti-apo A-I monoclonal antibodies isolate residual lipoproteins, excluding chylomicron remnants, allowing for their quantification [17]. Automated assays by Denka Seiken, employing enzymes and surfactants, have shown to measure RC concentrations that are significantly lower than calculated estimates, yet maintain a robust correlation (R^2^ = 0.73) [18]. Discrepancies between calculation and automated assay methods have been noted, particularly at higher RC levels. The automated assay has identified a subset of the population with elevated RC and low TG levels, conferring a slightly lower risk of all-cause mortality compared to the calculated method [19]. While calculated values may not precisely reflect true RC levels, this method is preferred for its convenience and cost-effectiveness, particularly in drug efficacy trials [20]. The potential of automated measurements to identify high-risk cardiovascular populations underscores the need to refine and promote this approach.

## 3. Correlations between RC and ASCVD Risk in Primary Prevention Population

### 3.1 RC and Cardiovascular Disease

Numerous epidemiological studies and meta-analyses have demonstrated a significant correlation between RC levels and the risk of ASCVD (Table 1, Ref. [18, 19, 21, 22, 23, 24, 25, 26, 27, 28, 29, 30, 31, 32]).

**Table 1.** **Epidemiological studies about correlations between RC and the risk of primary and secondary prevention population of ASCVD**.

Epidemiological Study	Cohort or author	Research type	Study populations	Follow-up period	Relationship between RC and ASCVD	Reference
Primary Prevention	JHS and FOCS	Prospective Study	4114 participants (JHS) and 818 participants (FOCS) without CHD	8 years	A 23% increase in CHD risk per 1-SD increase in RLP-C (HR: 1.23, 95% CI: 1.06–1.42, *p * < 0.01) after adjustment for cardiovascular risk factors in combined cohorts.	[21]
	ARIC, MESA and CARDIA	Prospective Study	17,532 ASCVD-free individuals	52.3 years	Log RC was associated with higher ASCVD risk (HR: 1.65, 95% CI: 1.45–1.89) after multivariable adjustment.	[22]
	CCHS and CGPS	Prospective Study	90,000 individuals from the Danish general population	22 years	Elevated nonfasting RC levels >58 mg/dL were associated with increased risk of IHD (HR: 2.4, 95% CI: 1.9–2.9) (*p* for trend <0.001) compared with RC levels <19.3 mg/dL after multivariable adjustment.	[18]
	CCHS, CGPS and CIHDS	Prospective Study	90,000 participants from Copenhagen	22 years	High IHD risk due to obesity can be explained partly by RC.	[23]
	CGPS	Prospective Study	106,216 individuals from CGPS	11 years	Elevated nonfasting RC levels ≥58 mg/dL were associated with increased risk of MI for normal weight individuals (HR: 2.0, 95% CI: 1.3–3.2), overweight individuals (HR: 1.9, 95% CI: 1.4–2.6), and obese individuals (HR: 2.3, 95% CI: 1.4–3.5) compared with RC levels <19 mg/dL.	[24]
	CGPS	Prospective Study	102,964 individuals from CGPS	14 years	Elevated baseline fasting RC levels ≥58 mg/dL were independently associated with higher ischemic stroke risk (OR: 1.99, 95% CI: 1.49–2.67) compared with RC levels <19 mg/dL.	[25]
	CGPS	Prospective Study	106,937 individuals from CGPS	15 years	Elevated non-fasting RC levels ≥58 mg/dL were associated with higher risk of PAD (HR: 4.8, 95% CI: 3.1–7.5) compared with RC levels <19 mg/dL.	[26]
Secondary Prevention	Takamitsu Nakamura *et al*.	Prospective Study	560 patients with coronary artery disease	36 months	RLP-C proved to be a significant predictor of future cardiovascular events (HR: 1.74, 95% CI: 1.31–2.32).	[27]
	Si Van Nguyen *et al*.	Prospective Study	190 patients treated with statins after ACS	70 months	Elevated fasting RC level ≥5.4 mg/dL was a significant risk factor for recurrent MACEs, independent of conventional risk factors (HR: 2.94, 95% CI: 1.40–6.18; *p * < 0.01).	[28]
	Xiangyu Xu *et al*.	Prospective Study	2832 patients with CAD who underwent successful PCI	15 months	Elevated fasting RC level >20 mg/dL was predictive of ISR occurrence with 77.9% sensitivity and 56.5% specificity (AUC: 0.705, *p * < 0.001, 95% CI: 0.648–0.762).	[29]
	Mohamed B Elshazly *et al*.	Prospective Study	5754 patients with coronary artery diseases	2 years	Elevated fasting RC levels ≥32.7 mg/dL were associated with higher incidences of 2-year MACEs compared with RC levels <17.8 mg/dL (23% versus 14%, log–rank *p * < 0.001).	[30]
	CIHDS	Prospective Study	5414 patients diagnosed with ischemic heart disease	7 years	Elevated RC can explain an 8% to 18% residual risk in all-cause mortality among IHD patients.	[19]
	A Varbo and BG Nordestgaard	Cross-sectional Study	142 patients with ischemic stroke	/	RLP-C levels higher than 5.56 mg/dL were approximately 2.5 times more likely to have ischemic strokes than controls.	[25]
	Yuki Fujihara *et al*.	Prospective Study	247 patients with CAD	38 months	Elevated fasting RC level >3.9 mg/dL was predictive of CVEs occurrence with 72.7% sensitivity and 58.4% specificity.	[31]
	Yi Song *et al*.	Cross-sectional Study	246 T2DM patients without PAD and 270 T2DM patients with PAD	/	Diabetic patients with remnant cholesterol levels >0.64 mmol/L had an increased risk of developing PAD (Sensitivity 71.9%, Specificity 64.6%).	[32]

JHS, Jackson Heart Study; FOCS, Framingham Offspring Cohort Studies; ARIC, Atherosclerosis Risk in Communities Study; MESA, Multi-Ethnic Study of Atherosclerosis; CARDIA, Coronary Artery Risk Development in Young Adults Study; CCHS, Copenhagen City Heart Study; CGPS, Copenhagen General Population Study; CIHDS, Copenhagen Ischaemic Heart Disease Study; 1-SD, one standard deviation; HR, hazard ratio; CI, confidence interval; OR, odds ratio; AUC, area under the curve; CHD, coronary heart disease; RC, remnant cholesterol; ASCVD, atherosclerotic cardiovascular disease; IHD, ischemic heart disease; MI, myocardial infarction; PAD, peripheral artery disease; CVEs, cardiovascular events; MACEs, major adverse cardiovascular events; RLP-C, remnant-like particle cholesterol; CAD, cardiovascular disease; T2DM, type 2 diabetes mellitus; ACS, acute coronary syndrome; PCI, percutaneous coronary intervention; ISR, in-stent restenosis.

In 2016, a combined analysis from the Jackson Heart Study (JHS) and Framingham Offspring Cohort Studies (FOCS) revealed that for each 1-standard deviation (1-SD) rise in remnant-like particle cholesterol (RLP-C), there was a 23% increase in CHD risk among participants free of coronary heart disease (CHD), after adjusting for established cardiovascular risk factors (hazard ratio [HR]: 1.23; 95% confidence interval [CI]: 1.06–1.42, *p* < 0.01) [21]. Similarly, Quispe *et al*. [22] conducted a prospective study involving 17,532 individuals without ASCVD. Over a mean follow-up period of 52.3 years, 2143 ASCVD events were recorded. Upon multivariate adjustment for LDL-C and apoB, the logarithm of RC was significantly associated with an elevated ASCVD risk (HR: 1.65; 95% CI: 1.45–1.89), suggesting that RC is an independent risk factor for ASCVD in primary prevention populations [22]. A Mendelian randomization study involving 73,513 participants from Copenhagen identified a 2.8-fold increase in the risk of ischemic heart disease (IHD) for each 1 mmol/L (39 mg/dL) increase in non-fasting RC, independent of changes in HDL-C [33]. Another Mendelian randomization study demonstrated that a doubling of non-fasting TG and calculated RC, attributed to *apolipoprotein A-V* (*apo A-V*) gene mutations, is associated with a 1.9-fold and 2.2-fold increase in the causal risk of myocardial infarction (MI), respectively [34]. Those mendelian randomization studies, which reduce the impact of confounding factors, provide stronger causal evidence compared to traditional observational studies.

Furthermore, a 22-year prospective study involving 90,000 participants from the Danish general population, including the Copenhagen General Population Study (CGPS) and the Copenhagen City Heart Study (CCHS), found that both non-fasting RC and LDL-C were positively associated with the risk of IHD and MI. Unlike LDL-C, non-fasting RC levels showed a linear relationship with all-cause mortality, while LDL-C levels exhibited a U-shaped association with mortality. This suggests that non-fasting RC may be a better predictor of all-cause mortality than LDL-C [18]. A recent study also suggested that RC might be associated with an increased risk of death from cancer or other non-cardiovascular causes, in contrast to LDL-C, which is predominantly linked to cardiovascular mortality [35]. This intriguing distinction calls for further research to explore the underlying mechanisms.

Additionally, in high-risk populations, such as individuals with obesity and diabetes, both TG and RC levels were independently associated with major adverse cardiovascular events (MACEs). Specifically, for every 10 mg/dL increase in non-high-density lipoprotein cholesterol (non-HDL-C) and RC levels, there was a corresponding 5% and 21% increase in MACE risk, respectively. The study further concluded that individuals with RC levels exceeding 30 mg/dL (0.78 mmol/L) face a higher cardiovascular risk, regardless of their LDL-C levels [23].

Varbo *et al*. [36] also discovered that genetically determined obesity raises the risk of IHD partially through elevated levels of non-fasting RC and LDL-C, as well as higher blood pressure. Their findings showed that the association between high RC levels and an increased risk of MI held across individuals with normal weight, overweight, and obesity. This reinforces the notion that RC is an independent risk factor for MI, irrespective of body weight [24].

### 3.2 RC and Cerebrovascular Disease

The influence of RC on vascular health extends to the risk of cerebrovascular accidents (CVA) and peripheral artery disease (PAD). Research has demonstrated a strong correlation between elevated RC levels and ischemic stroke and carotid artery abnormalities. Qian *et al*. [37] observed that with each 1 mmol/L (39 mg/dL) elevation in RC, there was a substantial 28% increase in the risk of abnormal mean carotid intima-media thickness (cIMT) and a 25% increase in the risk of abnormal maximum cIMT. These findings underscore the significance of RC as a predictive marker for early atherosclerotic changes in the carotid arteries [37]. A recent study has shown that even in the adolescent population, elevated levels of residual cholesterol are associated with increased intima-media thickness in the carotid arteries, an early marker of atherosclerosis [38].

In the CGPS, a large-scale prospective analysis of 102964 participants, researchers identified a positive association between RC concentrations and the risk of ischemic stroke [25]. Notably, the variability in RC levels (VIM, variance independent of the mean) was found to be significantly linked to a 9% increase in the risk of ischemic stroke for each 1-SD increase [39]. This may be due to RC’s propensity for auto-oxidation and cholesterol crystal formation within the subendothelial space, which can accelerate macrophage degeneration and promote plaque instability, increasing the likelihood of stroke [14, 40, 41]. Collectively, these findings demonstrate a strong correlation between elevated RC and ischemic stroke, particularly in large-vascular atherosclerosis-related stroke.

### 3.3 RC and Peripheral Vascular Disease

Recent research underscores the pronounced influence of RC on PAD, suggesting that its impact may even exceed that observed in coronary artery disease (CAD) and CVA. A comprehensive 15-year study of 106,937 individuals from the Copenhagen general population, using restricted cubic spline Cox models, showed that RC levels above 1.5 mmol/L were strongly associated with a higher risk of PAD compared to levels below 0.5 mmol/L (adjusted HR: 4.8; 95% CI: 3.1–7.5). Strikingly, elevated RC levels were associated with a 5-fold increased risk for PAD, a figure that surpasses the risks associated with MI and stroke [26].

## 4. Correlation of RC with Residual Risk of ASCVD in Secondary Prevention Populations

### 4.1 RC and Residual Risk of Cardiovascular Disease

Evidence from observational studies, genetic studies, and randomized controlled trials demonstrates that despite achieving optimal LDL-C levels using statins and non-statin drugs, a significant residual risk of MACEs persists in secondary prevention populations (Table 1).

One study involving 560 coronary artery disease patients undergoing lipid-lowering therapy with LDL-C levels below 100 mg/dL found that over a 36-month follow-up period, 40 cardiovascular events (CVEs) occurred. Stepwise Cox proportional hazard analysis identified RLP-C as a significant predictor of future cardiovascular events (HR: 1.74, 95% CI: 1.31–2.32) [27]. Similarly, a prospective study involving 190 patients with acute coronary syndrome (ACS) treated with statins, showed that high RC levels were a strong predictor of secondary cardiovascular events. These findings suggest that RC can be a valuable tool for risk stratification and treatment guidance following statin therapy for ACS [28].

RC has also been linked to in-stent restenosis (ISR) following percutaneous coronary intervention (PCI).

In patients who received drug-eluting stents (DES), RLP-C was an independent predictor of ISR, with an odds ratio (OR) of 4.154 (95% CI: 1.895–9.104; *p* < 0.01) for patients with diabetes mellitus and OR = 4.455 for those without diabetes (95% CI: 2.097–9.464; *p* < 0.01). A cut-off value of 0.515 mmol/L for RLP-C was identified as a predictive marker for ISR occurrence [29].

In patients with very low levels of LDL-C, the incremental increase in percentage atheroma volume (PAV) progression was 0.18% for each SD increase in RC (9 mg/dL) (95% CI: 0.07%–0.29%). Additionally, atherosclerotic plaque progression was seen when RC levels were >25 mg/dL in both statin- and proprotein convertase subtilisin/kexin type 9 (PCSK9) -treated patients [30]. Another study involving 5414 IHD patients found that elevated RC was a risk factor for all-cause mortality, explaining 8% to 18% of residual risk in mortality [19].

### 4.2 RC and Residual Risk of Cerebrovascular Disease

RC also has important predictive value for the development of cerebrovascular disease in secondary prevention populations for CAD. In a cross-sectional study of 142 ischemic stroke patients, those with RLP-C levels above 5.56 mg/dL were approximately 2.5 times more likely to experience an ischemic stroke than controls, suggesting that RLP-C is a risk factor for ischemic stroke, as corroborated by a study of the Copenhagen cardiac population [25, 31]. In addition, a high level of RLP-C emerged as the sole significant predictor positively correlated with large artery atherosclerosis (LAA)-subdivided stroke after adjusting for traditional risk factors and lipid parameters such as TG. The predictive value for LAA stroke was significantly higher than other subgroups [31].

In ischemic stroke patients, fasting RC levels were positively associated with subclinical carotid atherosclerosis. For every 1 mmol/L (39 mg/dL) increase in RC, there was a 28% increase in the risk of mean cIMT abnormality (OR: 1.28; 95% CI: 1.03–1.60) and a 25% increase in the risk of maximal cIMT abnormality (OR: 1.25; 95% CI: 1.01–1.54). Moreover, higher baseline RC levels (RC ≥0.43 mmol/L [16.60 mg/dL]) were independently associated with the composite outcome of severe disability and death (OR: 1.56; 95% CI: 1.01–2.39) [37]. A retrospective study included 587 patients undergoing computed tomography coronary angiography (CTCA) for suspected CAD with total coronary atherosclerotic load measured by CT-Leaman score (CT-LeSc). Mean RC levels were higher in patients with CT-LeSc >5 than in patients with CT-LeSc ≤5 (0.76 ± 0.36 mmol/L versus 0.58 ± 0.33 mmol/L, *p* = 0.01). RC significantly predicted atherosclerotic load adjusting for traditional risk factors (OR: 3.87; 95% CI: 1.34–7.55, *p* = 0.004) [38].

### 4.3 RC and Residual Risk of Peripheral Vascular Disease

Studies also suggest a significant relationship between RC and PAD, even in patients who have achieved optimal LDL-C levels. In one study of 247 patients with stable coronary artery disease and LDL-C levels below 70 mg/dL treated with statins, 33 cardiovascular events, including PAD requiring endovascular or surgical treatment or amputation, occurred over a mean follow-up of 38 months. Keplan-Meier analysis found that patients with higher levels of remnant-like particles (RLPs) (RLPs >3.9 mg/dL) were significantly more likely to have CVEs than patients with lower levels of RLPs (*p* < 0.01). RLP-C was found to be an independent predictor of future cardiovascular events after adjusting for traditional risk factors including TG and total apoB (HR: 1.62; 95% CI: 1.26–2.07, *p* < 0.01) [31]. Similarly, a cross-sectional study in diabetic patients showed that those with RC levels above 0.64 mmol/L had an increased risk of developing PAD (sensitivity 71.9%, specificity 64.6%). Further studies indicated that RC, independent of TG and HDL-C, was associated with the severity of PAD. Although this study did not consider non-fasting patients, its findings still provide valuable insights [32].

Despite achieving optimal LDL-C levels, the residual cardiovascular risk remains, possibly due to the duration and severity of abnormal LDL-C levels prior to treatment. Future research should consider incorporating the cholesterol-year score to better assess the long-term impact of cholesterol exposure on cardiovascular risk in primary and secondary prevention cohorts [42].

## 5. The Effects of Current Lipid-lowering Drugs on RC Levels

Current lipid-lowering therapies, including statins, combination regimens with ezetimibe, and PCSK9 inhibitors, have demonstrated significant efficacy in reducing RC levels. Emerging treatments such as omega-3 fatty acids, apoCIII inhibitors, pemafibrate, and bempedoic acid offer additional promise for managing residual cardiovascular risk, particularly in patients with suboptimal responses to statin monotherapy (Table 2, Ref. [7, 43, 44, 45, 46, 47, 48, 49, 50, 51, 52, 53, 54, 55, 56, 57, 58, 59, 60, 61]).

**Table 2.** **The effects of current lipid-lowering drugs on RC levels**.

Drug	Trial	Study population	Interventions	Media follow-up duration	Outcomes		Reference
					Effects on lipids/plaque	MACE incidence	
Statins (oral)	PREVAIL US Trial	328 participants with hyperlipidemia	pitavastatin 4 mg/d versus pravastatin 40 mg/d	12 weeks	Median RLP-C reduction: 34% versus 23%	/	[43]
	STELLAR trial	270 participants with hyperlipidemia	atorvastatin 80 mg/d and rosuvastatin 40 mg/d	6 weeks	Median RC reduction is similar: 58.7% versus 61.5%	/	[60]
Ezetimibe (oral)	Osman Ahmed *et al*.	40 patients with uncomplicated cholesterol gallstone disease	simvastatin 80 mg/d and ezetimibe 10 mg/d versus placebo, simvastatin 80 mg/d, ezetimibe 10 mg/d	4 weeks	Median RC reduction: 65% versus 51% (simvastatin) and 18% (ezetimibe)	/	[44]
	IMPROVE-IT study	18,144 patients after acute coronary syndrome	simvastatin 40 mg/d and ezetimibe 10 mg/d versus simvastatin 40 mg/d	7 years	Compared with simvastatin group, simvastatin–ezetimibe group showed 24% further lowering of LDL-C level	32.7% versus 34.7% (HR: 0.936; 95% CI: 0.89–0.99; *p* = 0.016)	[45]
PCSK9 monoclonal antibodies (subcutaneous)	Britt E Heidemann *et al*.	28 patients with FD	evolocumab 140 mg Q2W versus placebo	12 weeks	Compared with placebo, the relative reduction in RC levels was 44% (fasting status) and 49% (post fat load)	/	[46]
	Study 565 (NCT01288443)	60 non-FD patients with hyperlipidemia	alirocumab 150 mg Q2W versus placebo	12 weeks	Median RC reduction: 42.1% versus 4.4%	/	[47]
Lomitapide (oral)	M Cuchel *et al*.	29 patients with HoFH	a starting dose of 5 mg/day and then escalated to 60 mg/day	26 weeks	Median LDL-C reduction: 45.5% from baseline	/	[48]
	Blom DJ *et al*.	17 patients with HoFH	median lomitapide dose: 40 mg/day	126 weeks	Median LDL-C reduction: 50% from baseline	/	[49]
Omega-3 fatty acid (oral)	REDUCE-IT trial	8179 patients	IPE 4 g/d versus placebo	4.9 years	/	17.2% versus 22% (HR: 0.725, 95% CI: 0.68–0.83; *p * < 0.001)	[50]
	EVAPORATE trial	80 patients with one or more angiographic stenoses with ≥20% narrowing	IPE 4 g/d versus mineral oil placebo	18 months	Mean low-attenuation plaque progression: –17% versus +109%	/	[51]
	PROUD48 study	126 patients with hyperlipidemia	omega-3 fatty acid ethyl 4 g/d versus pemafibrate 0.4 mg/d	16 weeks	Median RC reduction: 28.9% versus 46.7%	/	[52]
	MARINE and ANCHOR studies	229 (the MARINE trial) and 702 (the ANCHOR trial) paticipants	icosapent ethyl: 4 g/day versus 2 g/day	12 weeks	Median RC reduction: the MARINE trial: 29.8% versus 14.9%; the ANCHOR trial: 25.8% versus 16.7%	/	[53]
Pemafibrate (oral)	PROMINENT trial	10,497 patients with hypertriglyceridemia and diabetes	pemafibrate 0.4 mg/d versus placebo	4 months	Median RC reduction: 25.6%	3.6% versus 3.51% (HR: 1.03; 95% CI: 0.91–1.15; *p* = 0.67)	[54]
Bempedoic acid (oral)	CLEAR Serenity trial	345 patients with hypercholesterolemia intolerable of statin therapy	Bempedoic acid 180 mg versus placebo	12 weeks	Non-HDL-C reduction: 17.9% (placebo-corrected change from baseline)	/	[61]
	CLEAR Tranquility trial	269 patients with hyperlipidemia intolerable of statin therapy	Bempedoic acid 180 mg/d and ezetimibe 10 mg/d versus placebo and ezetimibe 10 mg/d	12 weeks	Compared with placebo, the relative reduction in non-HDL-C levels was 23.6%	/	[55]
	CLEAR Outcomes study	13,970 patients with ASCVD or high risk of ASCVD but statin intolerance	Bempedoic acid 180 mg versus placebo	40.6 months	/	11.7% versus 13.3% (HR: 0.87, 95% CI: 0.79–0.96; *p* = 0.004)	[56]
ApoCIII Inhibitor (subcutaneous)	APPROACH trial	66 patients with FD	volanesorsen 300 mg/week versus placebo	3 months	Median TG reduction: 77% versus 18%	/	[57]
	Jean-Claude Tardif *et al*.	114 patients with hypertriglyceridemia	olezarsen versus placebo 50 mg every 4 weeks	25 weeks	Compared with placebo, the relative reduction in non-HDL-C levels was 20%	/	[58]
ANGPTL3 Inhibitor (subcutaneous)	DiscovEHR study	83 participants with heterozygous loss-of-function variants	evinacumab versus placebo		Median TG reduction: 76% (day 4) and median LDL-C reduction: 23% (day 15)	/	[7]
	GF Watts *et al*.	52 healthy participants	ARO-ANG3 versus placebo	85 days	Median TG reduction: 54% and median non-HDL-C reduction: 29%	/	[59]

LDL-C, low density lipoprotein-cholesterol; MACEs, major adverse cardiovascular events; RLP-C, remnant-like particle cholesterol; Non-HDL-C, non-high-density lipoprotein cholesterol; TG, triglyceride; ANGPTL3, angiopoietin-like protein3; PCSK9, proprotein convertase subtilisin/kexin type 9; RC, remnant cholesterol; FD, familial dysbetalipoproteinemia; HoFH, homozygous familial hypercholesterolemia; Q2W, every two weeks; IPE, icosapent ethyl; ASCVD, atherosclerotic cardiovascular disease; HR, hazard ratio; HDL, high-density lipoprotein; CI, confidence interval.

### 5.1 Effect of TC-lowering Drugs on RC Levels

Statins are effective at lowering RC by enhancing the hepatic uptake of TRLs through low-density lipoprotein receptors (LDL-R) and extrahepatic lipoprotein receptors (VLDL-R, very-low-density lipoprotein receptors) [3]. As a cornerstone of ASCVD prevention, statins are primarily used to reduce LDL-C levels. The PREVAIL trial highlighted the efficacy of statins in lowering RC levels, with pitavastatin 4 mg/day outperforming pravastatin 40 mg/day in ASCVD patients. Pitavastatin reduced RC by 13.6 mg/dL compared to 9.3 mg/dL with pravastatin, with median reductions in RLP-C of 34% and 23%, respectively [43]. Similarly, a post hoc analysis of the STELLAR trial revealed comparable RC reductions with atorvastatin 80 mg/day and rosuvastatin 40 mg/day (–58.7% versus –61.5%) [60]. While these studies did not directly link RC reduction to decreased ASCVD events, statins remain foundational in targeting RC to address residual risk.

Ezetimibe works by inhibiting intestinal cholesterol absorption through the Niemann-Pick C1-Like 1 receptor, thereby reducing cholesterol delivery to the liver, depleting hepatic cholesterol stores, and increasing cholesterol clearance from the blood [3]. Combining ezetimibe with statins results in a greater reduction in RC levels compared to statins alone. Simvastatin decreased RC by 51% (*p* < 0.001), ezetimibe by 18% (*p* < 0.001), and their combination by 65% (*p* < 0.001) [44]. The IMPROVE-IT study further illustrated that adding ezetimibe to simvastatin significantly reduced the incidence of MACEs in acute coronary syndrome patients compared to simvastatin monotherapy (32.7% vs. 34.7%; HR: 0.936; 95% CI: 0.89–0.99; *p* = 0.016) [45]. This benefit extended beyond LDL-C reduction, emphasizing the role of ezetimibe in RC management.

PCSK9, a pivotal protein modulating LDL-R expression, is targeted by PCSK9 monoclonal antibodies, which inhibit PCSK9 binding to LDL-R, thereby augmenting LDL-R availability, enhancing the removal of plasma LDL, and ultimately lowering circulating LDL-C levels [62]. PCSK9 inhibitors, including evolocumab (Repatha, Amgen Inc.) and alirocumab (Praluent, Regeneron Pharmaceuticals, Inc.), have been shown to markedly reduce LDL-C levels and cardiovascular events [63, 64, 65]. A study in 28 patients with familial dysbetalipoproteinemia (FD) treated with evolocumab 140 mg, in addition to standard lipid-lowering therapy, reported a 44% reduction in fasting RC and 49% reduction in postprandial RC after 12 weeks, with 89% of patients reaching their non-HDL-C goals [46]. Another post hoc analysis of alirocumab Phase II trials that enrolled 60 non-FD patients with hyperlipidemia, who were treated with alirocumab on top of stabilizing statin therapy for 12 weeks, showed a significant reduction in RC level of 42.1% compared to placebo (4.4%) [47]. PCSK9 inhibitors thus demonstrate substantial reductions in RC, both in patients with and without FD.

Lomitapide, a non-statin lipid-lowering agent approved for homozygous familial hypercholesterolemia (hoFH), works by selectively inhibiting MTTP, independent of LDL receptor (LDLR) function. While lomitapide has been shown to effectively reduce LDL-C levels in both the short and long term, its impact on RC levels remains unexplored, presenting a potential area for future research [48, 49].

### 5.2 Development of New TG-lowering Medications and Their Effect on RC Levels

#### 5.2.1 Omega3 Fatty Acid

Omega-3 fatty acids, especially eicosapentaenoic acid, have been shown to reduce cardiovascular risk, which can be attributed to their anti-inflammatory, anti-thrombotic, and plaque-stabilizing properties, as well as their ability to lower TG levels [66]. Notably, the REDUCE-IT trial demonstrated that icosapent ethyl at a dosage of 4 g/day significantly reduced the incidence of MACEs to 17.2% compared to 22.0% in the placebo group, independent of baseline triglyceride levels (HR: 0.75, 95% CI: 0.68–0.83; *p* < 0.001) [50]. The EVAPORATE trial further illustrated that icosapent ethyl could reduce low-attenuation plaque (LAP) by a remarkable 17% over 18 months, contrasting with a mineral oil placebo, which promoted LAP progression by 109%. This finding indirectly substantiates the hypothesis that omega-3 fatty acids can curtail ASCVD risk, given the vulnerability of low attenuation plaques [51]. However, trials such as ASCEND, STRENGTH, and OMEMI failed to demonstrate cardiovascular benefits, likely due to the use of lower doses or less purified forms of omega-3 fatty acids, warranting further research into their cardioprotective effects.

Regarding RC reduction, studies have confirmed the efficacy of omega-3 fatty acids. In a trial of 63 dyslipidemic patients on statin therapy, omega-3 fatty acid ethyl esters at 4 g/day significantly reduced apolipoprotein B-48 levels by 17.5% and RC by 28.9% over 16 weeks [52]. The MARINE trial, enrolling 229 hyperlipidemic participants, revealed that icosapent ethyl at 4 g/day could reduce RC levels more effectively than 2 g/day (29.8% versus 14.9%) [53]. The precise role of omega-3 fatty acid-induced RC reduction in diminishing residual ASCVD risk among statin-treated dyslipidemic patients remains to be elucidated, warranting additional trials to ascertain the extent to which reduced RC can mediate ASCVD risk reduction.

#### 5.2.2 Pemafibrate

Pemafibrate, a selective agonist of the PPARα nuclear receptor, may reduce plasma RC concentrations by inhibiting apolipoprotein C-II secretion, enhancing apolipoprotein A activation, and stimulating LpL [67]. The PROMINENT trial, encompassing 10,497 patients with hypertriglyceridemia and diabetes treated with statins, observed a 26.2% reduction in triglyceride levels and a 25.6% reduction in RC at 4 months; however, cardiovascular event incidence did not differ significantly from placebo-treated patients (HR: 1.03, 95% CI: 0.91–1.15; *p* = 0.67) [54]. Despite its effects on RC, the impact of pemafibrate on clinical outcomes requires further investigation.

#### 5.2.3 Bempedoic Acid

Bempedoic acid, an adenosine triphosphate citrate lyase (ACL) inhibitor, functions upstream in the cholesterol synthesis pathway by reducing intracellular acetyl coenzyme A production, leading to decreased cholesterol synthesis and increased LDL receptor activity [68]. Studies have indicated that daily administration of bempedoic acid 180 mg for 12 weeks led to a 17.9% reduction in non-HDL-C in hypercholesterolemic patients [69]. A combination of bempedoic acid (180 mg) and ezetimibe (10 mg) further lowered non-HDL-C by 23.6% compared to placebo after 12 weeks [55]. The CLEAR Outcomes study demonstrated that bempedoic acid reduced the risk of MACEs by 11.7% compared to 13.3% in the placebo group (HR: 0.87, 95% CI: 0.79–0.96; *p* = 0.004) in patients with ASCVD or high ASCVD risk who were statin-intolerant [56]. While its impact on RC levels remains to be explored, bempedoic acid shows promise in managing residual cardiovascular risk.

#### 5.2.4 ApoCⅢ Inhibitor

ApoCIII, a component of TRLs, elevates serum triglyceride levels by inhibiting LpL activity, reducing TG lipolysis, and enhancing hepatic TG synthesis [70]. Studies suggest that apoCIII loss-of-function heterozygotes exhibit lifelong 43% lower plasma RC levels, mediating a 37% reduction in ischemic vascular disease (IVD) risk and a 54% reduction in IHD risk [71]. Volanesorsen, an antisense oligonucleotide (ASO) targeting apoCIII mRNA, inhibits its translation or degrades the resulting complex. The APPROACH trial demonstrated that volanesorsen reduced TG levels by 77% compared to an 18% increase in the placebo group among patients with familial chylomicronemia syndrome and plasma TG >500 mg/dL [57]. However, it also highlighted a major side effect of volanesorsen—thrombocytopenia. Olezarsen, a next-generation ligand-conjugated apoC-III ASO, presents a more favorable safety profile. A study showed a dose-dependent reduction in triglyceride levels up to 60% and non-HDL-C levels up to 19% in response to Olezarsen treatment, administered as monthly doses ranging from 10 to 50 mg [58]. Consequently, apoCIII emerges as a promising therapeutic target. However, no studies have yet demonstrated the RC-lowering effects of apoCIII inhibitors. Large clinical outcome trials are needed to confirm their long-term efficacy and safety.

#### 5.2.5 Angiopoietin-like Protein 3 (ANGPTL3) Inhibitor

ANGPTL3, an inhibitor of LpL, hepatic lipase, and endothelial lipase, can reduce the lipolysis of plasma lipoprotein TG [72]. Inhibition of ANGPTL3 function can lower non-HDL-C levels. Recently, monoclonal antibodies such as evinacumab and transcriptional modulation by siRNA or ASO such as vupanorsen have been developed to inhibit ANGPTL3 function. The DiscovEHR study showed that evinacumab could reduce TG levels by up to 76% and LDL-C by up to 23% in participants with heterozygous loss-of-function variants [73]. A Phase 1 trial targeting ANGPTL3 with RNA interference demonstrated a dose-dependent reduction in TG levels by up to 54% and non-HDL-C levels by up to 29% [59]. However, the impact of ANGPTL3 inhibitors on reducing RC remains unclear and requires urgent further investigation.

## 6. Insights and Perspectives

A multitude of observational studies, genetic investigations, and randomized controlled trials have consistently established the independent predictive significance of RC across primary and secondary populations affected by cardiovascular, cerebrovascular, or peripheral vascular diseases. The development of lipid-lowering therapies that specifically target RC is anticipated to provide significant clinical benefits. However, several critical issues require further investigation:

Firstly, it is crucial to elucidate the mechanisms by which RC contributes to ASCVD to develop comprehensive prevention strategies that counteract its effects on systemic vasculature. Secondly, the identification of a precise biomarker for RC is essential to enable more accurate and cost-effective measurement methods. Thirdly, there are currently no established guidelines for normal or target RC levels. However, a threshold of 0.5 mmol/L (<19 mg/dL) has been adopted based on evidence from several large prospective cohort studies [26, 74]. Additionally, Langsted *et al*. [75] suggest that lowering RC to 0.8 mmol/L (32 mg/dL) may reduce the risk of recurrent MACE by 20% in secondary prevention settings. Further research is needed to refine these target values for greater precision. Lastly, thorough investigation of RC-targeted therapies is essential to ensure their safety, efficacy, and cost-effectiveness in preventing vasculopathy before RC can induce significant vascular damage.

## 7. Conclusions

Robust evidence from observational studies, genetic analyses, and clinical trials underscores the critical role of RC as an independent risk factor for a wide range of vascular diseases, including cardiovascular, cerebrovascular, and peripheral vascular conditions. Despite advances in lipid-lowering therapies, the persistence of residual cardiovascular risk highlights the need for interventions that specifically address RC. Future research should focus on unraveling the mechanisms through which RC drives atherosclerosis, developing precise and economical methods for RC measurement, and formulating targeted therapies that safely and effectively reduce RC levels. Addressing these challenges could substantially improve the management and prevention of ASCVD.
